# Inherited Unbalanced Reciprocal Translocation with 18p11.32p11.21 Tetrasomy and 9q34.3 Trisomy in a Fetus Revealed by Cell-Free Fetal DNA (cffDNA) Testing: Cytogenetic and Cytogenomic Characterization in Prenatal Diagnosis

**DOI:** 10.3390/genes15111464

**Published:** 2024-11-13

**Authors:** Carmela Ardisia, Luigia De Falco, Giovanni Savarese, Raffaella Ruggiero, Teresa Suero, Nadia Petrillo, Monica Ianniello, Roberto Sirica, Alessio Mori, Davide Cino, Maria Barbato, Giuseppina Vitiello, Antonio Fico

**Affiliations:** 1 Medical Genetics and Rare Diseases, S. Maria della Misericordia Hospital Perugia, 06129 Perugia, Italy; carmela.ardisia@ospedale.perugia.it; 2 AMES, Polidiagnostic Strumental Centre, Srl, 80013 Naples, Italy; giovanni.savarese@centroames.it (G.S.); raffaella.ruggiero@centroames.it (R.R.); teresasuero@alice.it (T.S.); nadia.petrillo@centroames.it (N.P.); monica.ianniello@centroames.it (M.I.); roberto.sirica@centroames.it (R.S.); mrolssa@gmail.com (A.M.); davidecino@gmail.com (D.C.); barbato.mary87@gmail.com (M.B.); centroames@libero.it (A.F.); 3 Fondazione Genetica per la Vita Onlus, 80132 Naples, Italy; 4 Department of Molecular Medicine and Medical Biotechnologies, Federico II University Hospital, 80131 Naples, Italy; dr.giuseppina.vitiello@gmail.com

**Keywords:** inherited unbalanced reciprocal translocation, non-invasive prenatal testing (NIPT), prenatal diagnosis, SNP-array analysis

## Abstract

Background/Objective: Balanced reciprocal translocations are structural chromosomal anomalies that involve a mutual exchange of segments between two non-homologous chromosomes with a consequent 50–80% risk of conceiving fetuses with unbalanced chromosomal anomalies. This study describes a 37-year-old woman, at 13 + 5 weeks of gestation, who is a balanced reciprocal translocation 46,XX,t(9;18)(q34;q11.2) carrier, with a high-risk non-invasive prenatal screening test, NIPT, for chromosome 18 aneuploidy. Methods: The highlighted aneuploidy was characterized with cytogenetic, FISH and SNP-array techniques. Results: Cytogenetic analysis, performed on flask-cultured amniocytes, indicated a 48,XX,+2mar karyotype on 50 metaphases. SNP array analysis showed a 15.3 Mb duplication of chromosome 18p (arr[hg19]18p11.32-p11.21(12,842-15,303,932)x4), consistent with a partial tetrasomy 18p, and a 926 kbp duplication of chromosome 9q (arr[GRCh37]9q34.3(140,118,286-141,044,489)x3), consistent with partial trisomy 9q. FISH analysis with a 9q34.3 probe was performed on flask-cultured amniocytes’ metaphases, highlighting the presence of a third signal on one of the two marker chromosomes (18p11.32-p11.21). Conclusions: The evidence of such partial aneuploidies suggests that different mutational events may be possible at meiotic segregation or probably post-meiotic segregation. The results obtained highlight the high sensitivity of the screening test, NIPT, with massive parallel sequencing, and the usefulness of cytogenetics, cytogenomics and molecular biology techniques, in synergy, to characterize and confirm positive NIPT results.

## 1. Introduction

Reciprocal balanced translocations (RBT) are structural chromosomal anomalies characterized by two breaks on two non-homologous chromosomes and by the mutual exchange of the affected segments, without loss or gain of genetic material. The RBT carrier is a phenotypically normal individual; however, the carrier may manifest a pathological phenotype if the translocation breakpoints involve a dominant gene or interfere with the expression of immediately neighboring genes [1]. Indeed, birth defects and/or neurodevelopmental disorders and mental disorders have been observed in approximately 27% of patients with de novo RBT [2], and all individuals with RBT are at risk of reproductive problems. Balanced reciprocal translocations have a frequency of 1/500 (approximately 0.5–0.2%) in the general population. They are diagnosed due to infertility, multiple abortions (RPL), stillbirth, or the presence of congenital anomalies and unbalanced karyotype at birth [3,4]. In 6.6% of couples with multiple abortions, one of the two members of the couple is a carrier of RBT [5,6,7]. The frequency of RBT is ~1.3% in infertile males [8,9]. Meiotic segregation of a balanced reciprocal translocation can produce balanced and unbalanced gametes as a consequence of five possible types of segregation. At meiosis I, after the gametogenesis, to achieve the pairing of homologues, the chromosomes arrange themselves forming a quadrivalent which undergoes segregation through five possible modes: alternate, adjacent-1, adjacent-2, 3:1 or 4:0 [1]. Alternating segregation produces balanced gametes. Adjacent-1, adjacent-2, 3:1, and 4:0 segregations produce unbalanced gametes. It is not easy to predict how an RBT will segregate because many factors are involved, including: (a) the type of chromosome involved and its size; (b) the size of the segments involved in the translocation; (c) the position of the breakpoints; (d) the genetic content; (e) the patient’s sex and other aspects [1]. It is likely that many segregation events that produce chromosomal imbalances may not be detected if they cause infertility or early embryonic death. RBT carriers produce approximately 10–20% euploid embryos [10,11]. Several studies [12] on meiotic segregation patterns have identified different elements that can influence the imbalances of reciprocal translocations, such as the sex and age of the carrier, the location of breakpoints, the type of chromosome and the quadrivalent structure [13,14,15,16,17]. Some studies have analyzed meiotic segregation in blastocysts using comprehensive chromosome screening (CCS) [14,16,18], demonstrating that segregation in blastocysts could be influenced by the presence of acrocentric chromosomes and terminal breakpoints, rather than the carrier sex [16], suggesting that, in the female group, there was a higher percentage of blastocysts with an unbalanced rearrangement than males. A 3:1 segregation appears to be more frequent in the female group and in the presence of an acrocentric chromosome (Acr-ch) [12]. In support of this observation, studies on gametes and embryos have highlighted that the sex of a carrier can influence the various modes of meiotic segregation and that the male carrier has a higher percentage of alternating segregation [19,20]. However, literature data reported that the proportions of adjacent-1, adjacent-2 or 3:1 segregations were significantly different in male and female carriers, while for alternating segregation, no difference was observed [12,14,21,22,23]. Although prenatal diagnosis can highlight the imbalances obtained from the malsegregation of a balanced translocation, the risk of miscarriage associated with these procedures cannot be excluded [24]. Imbalances in these translocations can also be detected by cell-free fetal DNA (cffDNA)-based screening (NIPT) as reported in several studies [25,26,27]. In these cases, the result is a partial deletion and duplication. Recently, Flowers et al. presented their experience on screening pairs of RBT carriers, analyzing whole genome cell-free DNA-based non-invasive prenatal screening (gw-NIPS) [28]. Here, we describe a prenatal case with unusual structural chromosome rearrangements evidenced by NIPT and characterized with cytogenetic and cytogenomic techniques.

## 2. Materials and Methods

### 2.1. cffDNA Isolation from Plasma and NIPT Analysis

For NIPS analysis, streck blood collection tubes were used to collect about 10 mL of peripheral blood from the pregnant women. Firstly, the blood sample was centrifuged at 1600× *g* for 10 min at 4 °C to separate the maternal plasma from peripheral blood cells. Then, 900 μL of plasma was used to extract cell-free DNA using the QIAamp DNA Blood MiniKit (Qiagen, Hilden, Germany) following the manufacturer’s protocol. NIPT analysis was performed using the VeriSeq NIPT Solution v2 pipeline (Illumina Inc., San Diego, CA, USA) according to which the pregnant woman can choose to have the results as Basic, with reporting for common autosomal trisomies and sex chromosomes aneuploidies (SCA) (if selected), or as genome-wide analysis if the detection of the genome-wide fetal anomalies were included (including rare autosomal aneuploidies and partial deletions and duplications ≥ 7 Mb) [29,30]. Sample results were classified using the VeriSeq NIPT Solution v2 Assay Software (https://emea.support.illumina.com, accessed on 15 November 2021) (Illumina Inc., San Diego, CA, USA) and analysis of “raw data” was performed, as reported previously [29,31].

### 2.2. DNA Extraction, QF-PCR and Karyotype Analysis

Genomic DNA was extracted from the amniocyte using the QIAamp DNA Blood Mini Kit (Qiagen, Hilden, Germany)). The quantitative fluorescent polymerase chain reaction test for rapid aneuploidy detection was performed, using the Devyser Compactv3 QF-PCR kit (QF-PCR; Devyser Compactv3, Devyser, Stockholm, Sweden) as previously described [32]. GTG-banding analysis of amniotic fluid was performed in established cell culture following standard laboratory protocols. Then, 50 metaphases were analyzed with the CytoVision software (CytoVision Version 7.6, Leica Biosystems, Richmond, IL, USA CytoVision).

Fluorescent in situ hybridization (FISH) was performed on flask-cultured amniocytes’ metaphases. An RP11-644H13 FISH Probe (GRCh37/hg19: 140535110-140719726, Empire Genomics Green) (9q34 locus-specific probe, labelled in red), was used to highlight the partial trisomy 9q34.3). FISH slides were examined by using the LEICA DM5500 B fluorescent microscope (Tokyo, Japan) and analyzed with the CytoVision software (CytoVision Version 7.6, Leica Biosystems, Richmond, IL, USA CytoVision).

Single Nucleotide Polymorphism (SNP array) analysis was performed using the HumanCytoSNP-12v12.1 kit following the manufacturer’s protocol https://emea.support.illumina.com/, accessed on 25 November 2021). Stained BeadChips were scanned using a HiScan (Illumina, Inc., San Diego, CA, USA). GenomeStudio and Bluefuse Multi Software v.4.5 (Illumina, Inc., San Diego, CA, USA) were used to perform, respectively, the generation of data and analysis of the results. All CNVs > 100 Kb were interrogated. All results were reported according to GRCh37 (hg19) assembly.

## 3. Case Report and Results

A 37-year-old Caucasian woman came to the attention of the Ames Laboratory for NIPT testing at 13 + 5 weeks of gestation. The pregnant woman reported a history of multiple miscarriages in the maternal branch and that her brother, with a cleft palate and left preauricular appendix, died a few days after birth due to cardio-respiratory failure.

**Figure 1:**
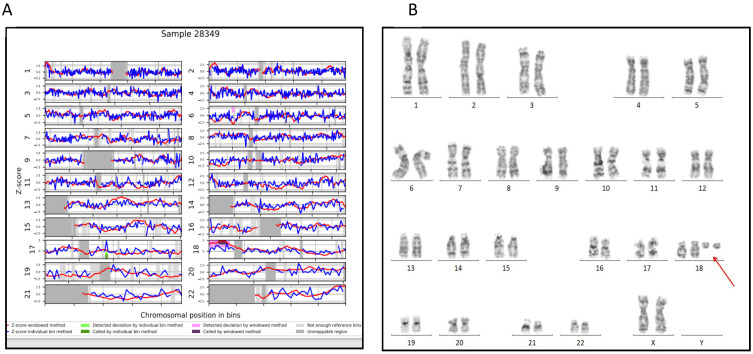
(**A**). NIPT results: WISECONDOR plot showing the abnormal NIPT result with a duplication of the short arm of chromosome 18. (**B**). GTG banding on amniotic fluid cultured cells’ metaphases showed a 48,XX,+2mar karyotype. Red arrow indicates the two markers.

Moreover, her mother had two miscarriages for unknown causes. The pregnant woman and her husband, following a repeated abortion, performed a constitutional karyotyping at another genetic laboratory. The husband’s karyotype was normal, while the woman’s karyotype highlighted a balanced reciprocal translocation involving the long arm of chromosome 9 and the long arm of chromosome 18: 46, XX, t(9;18) (q34;q11.2). The pregnant woman had a first pregnancy, which terminated in IVG, because of an unbalanced t(9;18), and a second pregnancy ended in a miscarriage. The couple had a healthy child with a balanced reciprocal translocation t(9;18), completely similar to the maternal one. For the pregnancy described, the first-trimester ultrasound findings, at 12 + 4 weeks, were normal with a nuchal translucency (NT) of 1.9mm. Threats of abortion were reported in the first few weeks and treated with progesterone therapy for a month. The woman decided to perform the NIPT screening test for previous miscarriages. NIPT analysis showed a high risk for duplication on chromosome 18: dup(18)(p11.32q11.2) and a fetal fraction of 7% (Figure 1A). In addition, the observed LLR scores for trisomy 18 in the “raw data” were lower than expected in the chromosomes 18 with trisomy 18 (internal validated data) and the case was considered a complex chromosomal abnormality. In light of this result, the woman decided to confirm the aneuploidy T18, undergoing amniotic fluid sampling at 16 + 0 weeks. QF-PCR for the trisomies 13, 18, 21 and sex chromosomes’ aneuploidies showed partial trisomy of the short arm of chromosome 18.

The fetal G-banding karyotype analysis, performed on flask-cultured amniocytes, highlighted a karyotype 48,XX,+2mar on 50 metaphases (Figure 1B). Cytogenomic characterization was thus performed to define the nature of the markers using the SNP-array test.

**Figure 2:**
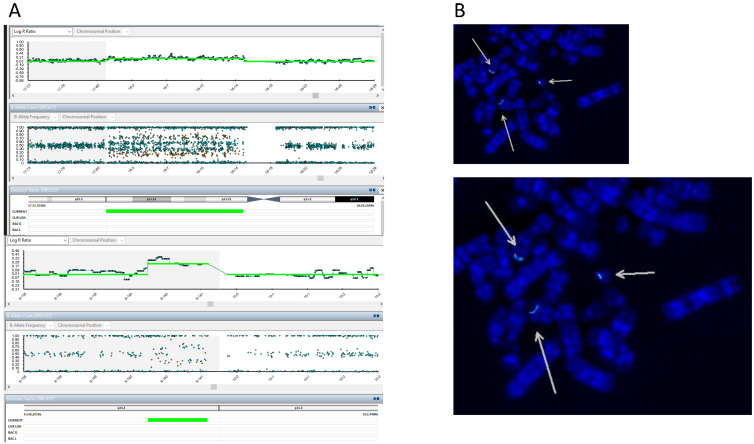
(**A**). SNP-array analysis on uncultered amniocytes showed a 15.3 Mb duplication of the chromosome 18p (arr[hg19]18p11.32-p11.21(12,842-15,303,932)x4) and a 926 kbp duplication of chromosome 9q (arr[GRCh37]9q34.3(140,118,286-141,044,489)x3). Stained BeadChips were scanned using a HiScan System (Illumina). (**B**). FISH analysis was performed with the RP11-644H13 FISH Probe (GRCh37/hg19: 140535110-140719726, Empire Genomics Green) on flask-cultured amniocytes’ metaphases. A positive 9q34.3 green signal indicated by the arrow is found at the terminal segment of one of the two marker chromosomes (18p11.32-p11.21) (LEICA DM5500 B fluorescence microscope) and analyzed with the CytoVision software (CytoVision, AB Imaging).

SNP array analysis on uncultured amniocytes showed a 15.3 Mb duplication of the chromosome 18p (arr[hg19]18p11.32-p11.21(12,842-15,303,932)x4) consistent with a partial tetrasomy 18p and a 926 kbp duplication of chromosome 9q (arr[GRCh37] 9q34.3(140,118,286-141,044,489)x3) consistent with a partial trisomy 9q (Figure 2A). FISH analysis was performed on flask-cultured amniocytes’ metaphases and highlighted the presence of a third signal on one of the two marker chromosomes (18p11.32-p11.21) (Figure 2B).

An ultrasound scan, performed at 19 weeks, showed an empty right renal lodge with a kidney located in the ipsilateral pelvis. After the genetic counseling, the couple decided to terminate the pregnancy at 19 + 4 weeks. Unfortunately, no autopsy was performed on the fetus.

## 4. Discussion

Reciprocal translocations are the most common structural chromosomal abnormalities resulting from the mutual exchange of non-homologous chromosome segments. They are present in 0.14% of the neonatal population and in 0.6% of infertile couples [33,34]. Carriers of a balanced reciprocal translocation are at risk of producing unbalanced gametes which, after being fertilized, can lead to the formation of an embryo with an unbalanced karyotype due to the presence of partial trisomies and/or monosomies [35]. In the present study, we described a fetal karyotype, following a high-risk NIPT test for trisomy 18, with a partial tetrasomy 18p and a partial trisomy 9q, originating in part from malsegregation of a balanced maternal reciprocal translocation (t(9;18)(q34; q11.2)) and whose evidence suggests that different mutational events may be possible. Meiotic segregation of a balanced reciprocal translocation can lead to the formation of balanced and unbalanced gametes, as a consequence of five possible types of segregation: alternate, adjacent-1, adjacent-2, 3:1 or 4:0 (both at meiotic segregation) [36]. In light of the results obtained in our case, it is conceivable to hypothesize that the gametogenesis of the female carrier of the balanced reciprocal translocation t(9;18) occurred with 3:1 segregation. We hypothesize that during 3:1 maternal segregation, a gamete with 24 chromosomes could be formed, due to the presence of an additional chromosome marker: der(18)t(9;18)(q34;q11.2). In our case, the maternal gamete, fertilized by a presumably normal paternal gamete, generated a zygote with 47 chromosomes with an additional chromosome marker: der(18)t(9;18)(q34;q11.2). Consequently, it may follow that the zygote thus formed should have a partial trisomy 18p11.32p11.21 region and 9q34.3 region. FISH analysis with the 9q34 telomeric probe highlighted the presence of a signal on the terminal segment of one of the two marker chromosomes, identified as 18p. From the tests performed and possible at the time of the genetic investigations, however, it was not easy to find proof of the origin of the second marker, der(18)(p11.32p11.21), also given the difficulty of being able to further study the biological samples of the two parents. Therefore, it is only conceivable that: (a) a nonallelic homologous recombination (NAHR) may have occurred during maternal meiosis; (b) a mitotic non-disjunction in the embryo with subsequent NAHR; (c) a possible gonadal mosaicism, both maternal and paternal. In a study, meiotic segregation modes of structural rearrangements highlighted in blastocyst biopsies, obtained from individuals carrying balanced reciprocal and balanced translocations, obtained by PGT, were analyzed, and it was seen that there is a greater incidence of 3:1 segregation in the translocations in which there are involved acrocentric chromosomes [18], which was consistent with studies from the Lim [37] and Ye [23] groups. The authors also take into consideration the importance of an interchromosomal effect (ICE) which could disturb the correct pairing and disjunction of other chromosomes during meiosis I and, as a consequence of this, could lead to chromosomal numerical anomalies which are not present translocation [38]. This study demonstrated that non-translocated chromosomal abnormalities were significantly higher in translocations not involving acrocentric chromosomes compared to translocations with acrocentric chromosomes, suggesting that ICE may be influenced by the chromosome types involved in the translocation. What the authors report could help us interpret and explain the presence of the second chromosomal marker in the fetal karyotype. In almost all the cases described in the literature, tetrasomy 18p was derived from an additional isochromosome of the short arm of chr18, (i(18p)), whereas our case presents a partial tetrasomy of the short arm of chromosome 18 (p11.3p11.2). Tetrasomy 18p syndrome (OMIM#614,290) is a very rare chromosomal disorder with a prevalence between 1/140,000 and 1/180,000 [39] and affects both sexes equally. Most tetrasomy 18p cases are “de novo” and maternal meiosis II nondisjunction [40] origin of trisomy 18p is a rare condition [41,42,43,44]. Individuals with tetrasomy 18p clinically present developmental delay, cognitive impairment, microcephaly, hypertonia, strabismus, and scoliosis/kyphosis [39]. The prenatal ultrasound diagnosis of tetrasomy 18p syndrome is difficult due to the absence of specific morphologic features. Therefore, ultrasound examination in synergy with a prenatal genetic test is an effective method for the diagnosis of tetrasomy 18p. In the case we are describing, amniocentesis was performed to confirm the positive NIPT result. Actually, the woman, as a carrier of an apparently balanced reciprocal translocation, should have chosen an invasive genetic test, but due to her personal and family history of repeated miscarriage, she preferred a non-invasive prenatal screening test, NIPT, despite being aware of the problem of fetal-placental mosaicism that this test may present. However, the ultrasound performed during the second trimester of pregnancy did not highlight any clinical signs attributable to chromosomal aneuploidy, perhaps due to the early gestational age or the limitations of ultrasound. Furthermore, the ultrasound examination did not reveal any signs relating to partial trisomy 9q34. Indeed, individuals described with dup 9q34 present with mild psychomotor retardation, low birth weight, normal length at birth and poor initial feeding and growth, joint contractures, long thin limbs, evident arachnodactyly, abnormal thumb implantation, increased space between the first and second toes and an excess of digital creases, erythema and heart murmurs, ptosis and strabismus, dolichocephaly, facial asymmetry, narrow horizontal palpebral fissures, microphthalmia, prominent nasal bridge, small mouth, thin upper lip with downturned corners, and mild retrognathia [45,46,47,48]. Other reports of cases of trisomy 9q34.1qter were the result of unbalanced segregation of reciprocal translocations and therefore associated with some other chromosome imbalance [49,50,51,52,53,54]. In light of the results obtained and after genetic counselling the woman decided to terminate the pregnancy. Unfortunately, no autopsy was performed on the fetus; thus, we cannot add partial phenotype to this report. The NIPT test, despite being a screening test, in the case described, proved to be highly sensitive, highlighting the possible presence of an aneuploidy of chromosome 18, confirmed by the cytogenomic results. In addition to trisomy 13, 18 and 21, NIPT can detect structural chromosomal abnormalities, such as deletions and duplications [55,56], although in our case, the 926 kbp duplication was not identified by NIPT due to size limitations. Our case confirms the high sensitivity of NIPT, based on genome-wide massive parallel sequencing for chromosome 18, in that a triplicated p-arm (four copies) of chromosome 18 corresponding to an NCV of approximately 40% increased in respect to the fetal fraction of DNA compared to that of a full trisomy 18, giving significant risk for chromosome 18 aneuploidy by NIPT. Chromosomal microarray and FISH on amniocytes confirmed that the increased NCV for chromosome 18 was due to fetal tetrasomy 18p and not to maternal mosaicism. In addition, a 926 kbp duplication of chromosome 9q was found, which suggests that different mutational events may be possible, probably at segregation or post-meiotic time. The use of the cytogenetics, cytogenomics and molecular biology techniques, in synergy, helps to characterize and confirm the positive NIPT results.

## 5. Conclusions

In this case report, we present a fetus with an unexpected structural chromosomal abnormality suspected by routine basic non-invasive prenatal testing (NIPT) including trisomy 13, 18 and 21 in a high-risk first-trimester pregnancy. The evidence of such partial aneuploidies suggests that different mutational events may be possible, at meiotic segregation and/or perhaps post-meiotic time. This case confirms the high sensitivity of NIPT, based on genome-wide massive parallel sequencing for common aneuploidies, as well as for the detection of sub-chromosomal abnormalities. The use of the cytogenetics, cytogenomics and the molecular biology techniques, in synergy, help to characterize and confirm the positive NIPT results.

## Data Availability

All data generated or analyzed during this study are included in this published article. Protocols and deidentified, aggregated data that underlie the results reported in this article are available for non-commercial scientific purposes upon reasonable request from the corresponding author. For privacy reasons raw data are not publicly available.
